# The relationship between microRNAs and COVID-19 complications

**DOI:** 10.1016/j.ncrna.2024.08.007

**Published:** 2024-08-22

**Authors:** Abdollah Kebriaei, Reza Besharati, Hasan Namdar Ahmad Abad, Shahrzad Havakhah, Mahsa Khosrojerdi, Amir Azimian

**Affiliations:** a Department of Pathobiology and Laboratory Sciences, Faculty of Medicine, North Khorasan University of Medical Sciences, Bojnurd, Iran; b Department of Physiology, Faculty of Medicine, North Khorasan University of Medical Sciences, Bojnurd, Iran; c Department of Immunology and Allergy, Faculty of Medicine, Mashhad University of Medical Sciences, Mashhad, Iran

**Keywords:** COVID-19, microRNA, Obesity, Diabetes, Neurological disease, Heart disease

## Abstract

Over the past three years, since the onset of COVID-19, several scientific studies have concentrated on understanding susceptibility to the virus, the progression of the illness, and possible long-term complexity. COVID-19 is broadly recognized with effects on multiple systems in the body, and various factors related to society, medicine, and genetics/epigenetics may contribute to the intensity and results of the disease. Additionally, a *SARS-CoV-2* infection can activate pathological activities and expedite the emergence of existing health issues into clinical problems. Forming easily accessible, distinctive, and permeable biomarkers is essential for categorizing patients, preventing the disease, predicting its course, and tailoring treatments for COVID-19 individually. One promising candidate for such biomarkers is microRNAs, which could serve various purposes in understanding diverse forms of COVID-19, including susceptibility, intensity, disease progression, outcomes, and potential therapeutic options. This review provides an overview of the most significant findings related to the involvement of microRNAs in COVID-19 pathogenesis. Furthermore, it explores the function of microRNAs in a broad span of effects that may arise from accompanying or underlying health status. It underscores the value of comprehending how diverse conditions, such as neurological disorders, diabetes, cardiovascular diseases, and obesity, interact with COVID-19.

## 1 Introduction

COVID-19 is an infection triggered by the *SARS-CoV-2* [1]. In roughly 80 % of instances, *SARS-CoV-2* leads to a relatively symptom-free or smooth superior respiratory tract infection resembling the flu. In comparison, in the residual 20 % of cases, it can progress to pneumonia, resulting in an intensive or severe form of the illness necessitating hospitalization [2]. Given its high virulence and ability to spread rapidly, the virus has been a primary concern for the medical society at the outset of the COVID-19 pandemic [3]. Apart from the virus's virulence and pathogenicity, researchers have recognized multiple individual risk factors that influence the range of intensity of COVID-19 infections across those subjected. These factors include age, underlying health conditions, sex, socioeconomic status, ethnicity, etc. [4]. Anyway, it has become evident that both viral characteristics and host conditions play a crucial role in determining a patient's susceptibility to SARSCoV-2 infection [5]. The variety in human genetics contributes to the variability in the immune reaction to SARS-CoV-2, which explains the broad range of symptoms and differing consequences observed in individuals infected with *SARS-CoV-2* [6]. In many manuscripts, connections were established between genetic elements, uncommon mutations, and inherent deficiencies in the immune system linked to COVID-19 [7]. This presents an opportunity to explore novel interventions at the cellular level and develop drug treatments to correct gene dysregulation in affected patients. Various therapeutic approaches, including anti-inflammatory, anticoagulant, immunotherapeutic, and antiviral strategies involving antibodies, have been employed to reduce fatality related to COVID-19 disease [8].

**Fig. 1:**
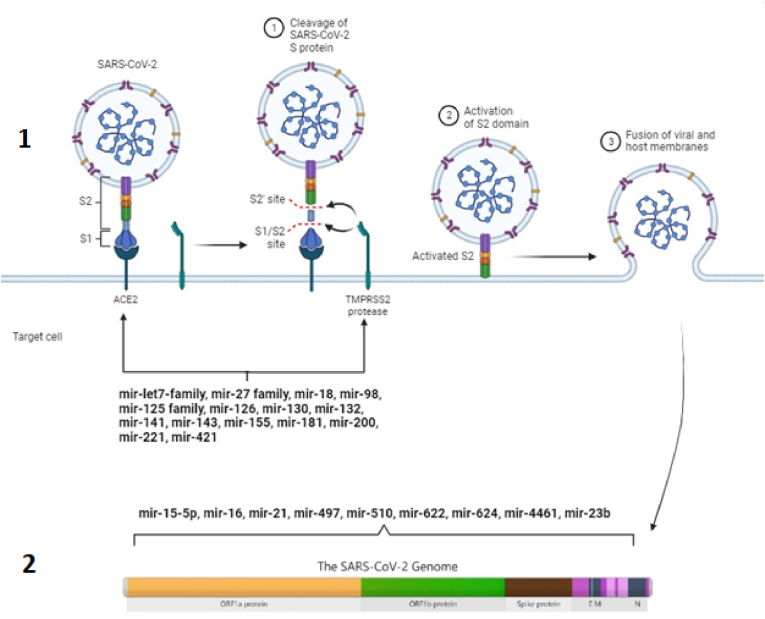
MicroRNAs and SARS-CoV-2. The role of microRNAs in various stages of COVID-19 pathogenesis. Part 1, shows the effect of microRNAs on the primary attachment stage and part 2 shows the direct effect of microRNAs on *SARS-CoV-2* genome.

Novel researches have introduced another innovative treatment target – microRNAs as an effective method to deactivate the SARS-CoV-2. This approach aims to reduce the risk of severe COVID-19 and restore common biological operations or adjust genetic responses. MicroRNAs are innate, small, single-stranded RNAs that govern protein production by inhibiting the specific mRNAs. They are crucial in determining cell recognition within expansion and harmonizing cellular activities entire a cell's lifespan [9]. These non-coding small RNAs, typically 21–23 nucleic acids in length, can influence nearly 30 % of the genome and participate in different pathways, containing those related to cell growth, death, lipid variation, and stress persistence [10]. MicroRNAs also have a significant role in the interaction between *SARS-CoV-2* and its host, impacting viral replication. They achieve this by binding to specific regions of viral RNA, such as the 3′- or 5′-UTR, or by influencing the production of molecules involved in the *SARS-CoV-2* lifetime, includes, transmembrane protease serine type-2 (TMPRSS2), angiotensin-converting enzyme-2 (ACE2), S proteins and Nsp12 [11]. [Fig. 1].

Previously identified microRNAs have a role in the development of viral infections, especially in coronaviruses, by participating in both the immune response and the expression of viral proteins [12]. Moreover, microRNAs are involved in fine-tuning cellular functions, which is crucial for developing adaptive and innate immune cells [13]. Several of these microRNAs can be evaluated as essential modulators of inflammatory factors [14]. Furthermore, it has been observed that host microRNAs may play a part in the cytokine flood correlated with COVID-19 [13]. They also have a role in the suppression of *SARS-CoV-2* activity [14]. It is important to note that the production of microRNAs can be controlled by human cells, which can lead to the blockage of virus replication through complex mechanisms. These mechanisms may involve regulating innate immunity pathways or direct interactions with the virus [15]. Additionally, the expression of host genes can be influenced by microRNAs encoded by the virus, potentially having a detrimental impact on the host's antiviral immune response [15]. A single microRNA can regulate several genes and, vice versa [16]. Moreover, since the microRNAs are encoded mainly on the X chromosome, they are often expressed at higher levels in women than men. Klein et al. reported the differences between male and female COVID-19 severity and deaths in the United States. They assumed these differences are related to the level of expression and activity of ACE2-receptor and a higher expression of X-Chromosome-related genes such as antiviral immunity and microRNAs genes [17].

We believe that the four-year period since the arrival of *SARS-CoV-2* provides a related starting matter to consider potential document-based diagnostic and prognostic indicators, such as free microRNAs, for COVID-19 convolution. One perspective to contemplate is regarding COVID-19 as a primary instigator of a multi-organ disorder. Alternatively, it can be viewed as an event that activates certain pre-existing conditions that may have been initiated but not yet manifested clinically, a concept particularly evident in neurodegenerative diseases. Furthermore, there is the potential to regard *SARS-CoV-2* as an integral component of the exposome, rather than isolating it from other influences, demographic factors, and changes in health and physiological situations, such as those associated with obesity. The objective of this manuscript is to emphasize the fundamental discoveries concerning microRNA as possible indicators for various trajectories and outcomes of COVID-19, particularly in the basis of multiple health conditions, with specific attention to Diabetes, Heart disease, obesity and neurological disorders.

## 2 Materials and methods

In this research, we employed a comprehensive method to review the existing literature on microRNA in the context of COVID-19 and condense the information to guide the following studies. This comprehensive overview was carried out in five systematic stages [1]: the recognition of pertinent published articles [2], the selection of relevant studies [3], the recognition of common subjects of interest [4], the presentation of the primary discoveries based on these subjects, and [5] the synthesis of the findings. In the context of this review, we examined and analyzed 84 articles. Next, we pinpointed the most repeatedly explored subjects within these studies. These areas of focus encompass: (i) the involvement of microRNAs in COVID-19 disease, (ii) the role of microRNAs in modulating the cell protein ACE2, (iii) the impact of microRNAs on the cellular mediator neuropilin-1 (NRP1), (iv) the influence of microRNAs in obesity, (v) the role of microRNAs in neurological diseases, and (vi) diabetes. In the third phase, we detailed the primary findings within each of these categories, elucidating how microRNAs regulate cell functions (including upregulation and deregulation) and protein production (including overexpression and suppression) in individuals with both COVID-19 and multiple health conditions. Lastly, in the concluding step, we summarized the significance of gene overexpression and suppression, as well as their upregulation and deregulation, to underscore the importance of these findings in the COVID-19 therapy and to provide insights for future research in drug development.

## 3 Results and discussion

### 3.1 microRNAs role in COVID-19

In various studies researchers found that microRNAs can be effective on *SARS-CoV-2* infection by affecting viral replication, translation, and the regulation of host gene expression [18]. Adan and Demirci has identified 67 distinct human microRNAs that specifically affect the S-protein of the *SARS-CoV-2* and disturbs primary attachment and entrance of virus to the host cells [18,19]. In Yang et al.'s study, it was proposed that specific microRNAs might potentially suppress the production of proteins linked to the *SARS-CoV-2* infection cycle, including TMPRSS2, ACE2, S-proteins, and Nsp12(11). Sometimes, the viral proteins can impair host immunity with their own microRNAs and specific proteins [20]. The roles of TMPRSS2 and ACE2 in *SARS-CoV-2* infection have been previously elucidated. These proteins plays a role in attachment, maturity of surface proteins and entrance to the host cells [21]. Notably, the virus relies on TMPRSS2 and ACE2 to enter host cells and perpetuate the infection, although it is essential to remember that not all tissues and cells express these proteins [21]. In some studies, researchers have evaluated and compared microRNAs in blood mononuclear cells of *SARS-CoV-2* positive patients and negative control groups. They identified several microRNAs includes miR-155-5p, miR146a-3p, and miR-29a-3p, in *SARS-CoV-2* positive patients, but not in control group. These microRNAs are promising indicators for recognizing COVID-19 from individuals without the virus. However, we have to accept that there are cheaper and easier ways to diagnose this disease. Furthermore, they found that miR-146a-3p, let-7b-3p, and miR-29a-3p could serve as potential markers to differentiate post-acute COVID-19 from healthy individuals. Additionally, miR-146a-3p and miR-29a-3p may be valuable biomarkers for distinguishing between acute and post-acute COVID-19 [[22], [23], [24]]. Calderon-Dominguez et al. observed that special microRNAs, namely miR-98-3p, miR-32-5p, miR-1246, and miR-423-3p, exhibited higher expression levels in critically ill COVID-19 patients compared to individuals who tested negative for SARS-CoV-2. Notably miR-1246 and miR-32-5p were remarkably increased in COVID-19 patients compared to asymptomatic patients [25]. These data suggest that specific microRNAs could potentially serve as biomarkers for assessing the severity of COVID-19 [26,27]. Additionally, in other study, the subsequent microRNAs were proved elevated in acute phase COVID-19 patients in comparison to a healthy persons: miR-21 (related to fibrosis), miR-155 (linked to inflammation), and miR-499 and miR-208a (specific to the heart muscle) [28]. In the initial cohort study, they found that miR-126 exhibited protective effects on endothelial cells and contributed to reducing damage [28,29]. Additionally, it was observed that after adjusting for age and sex, miR-221, miR-146, and miR-155 were particularly important in identifying acute COVID-19 patients. Notably, miR-122-3p can target various critical components such as tumor necrosis factor-alpha (TNF-α), toll-like receptors (TLRs), interleukin 8, interleukin 6, and transcription factors like NF-kB, all of which play crucial roles in the immune response [30,31]. McDonald et al., based on RNA sequencing data from COVID-19 patients, established that miR-2392 was the sole microRNA that showed increased expression in these patients [32]. Moreover, miR-2392 has a role in the enhancement of inflammation, hypoxia, and glycolysis. The urine and blood level of miR-2392 in COVID-19 patients is related to the viral load and makes it a potential prognostic biomarker [32]. Mishra et al. have evaluated the microRNAs in diabetic patients in the COVID-19 era and found that miR-133a is a suitable target for evaluation the role in heart failure in diabetic COVID-19 patients [33]. Except blood, urine, plasma and pulmonary fluids, microRNAs can be secreted in saliva. Saulle et al. have checked and compared the SARS-CoV-2 load, cytokines, microRNAs and neutralizing activity of saliva and plasma in mild and severe COVID-19 patients. They found downregulation of microRNAs let-7a-5p, let-7b-5p, and let-7c-5p and upregulation of miR-23a and b and miR-29c and also some immunomodulatory microRNAs. Evaluation of these microRNAs could be useful to predict disease outcomes and progression [34]. Using computational models to predict the role of microRNAs in various clinical and subclinical health conditions can offer a cost-effective strategy for diagnostic and therapeutic biomarker development. In COVID-19 research, many studies have employed advanced machine-learning models to pinpoint potential starting points and streamline the selection of research targets. These models rely on internal loops or asymmetric bulges within pre-microRNAs [35,36], which are pivotal in the identification process. However, it is crucial to note that developing clinically relevant biomarkers is not feasible without access to human cell lines and clinical samples [35] [Table 1].

**Table 1:** Selected microRNAs differentially expressed in patients with COVID-19 and patients with COVID- 19 and conditions: Diabetes, obesity, cardiovascular diseases or neurologic disorders. ↑upregulated, ↓downregulated.

COVID-19	ref	Diabetes	ref	Obesity	ref	Cardiovascular Diseases	ref	Neurologic disorders	ref
miR-423 ↑	[25]	miR-21↑	[28]	miR-146a↓	[71]	miR-133a↓	[33]	miR-31↓	[95]
miR-146a↑	[22]	miR-34a↑	[55]	miR-126↓	[28]	miR-15b-5p↓	[33]	miR-143↓	[107]
miR-627↑	[113]	miR-146a↑	[109]	miR-421↑	[65]	miR-30e-3p↓	[33]	miR-558↓	[107]
miR-208a↑	[28]	miR-384↓	[114]	miR-200c↑	[65]	miR-1↓	[13]	let-7a↓	[93]
miR-2392↑	[32]	miR-122↓	[30]	miR-3909↑	[67]	miR-133↑	[33]	let-7f↓	[93]
miR-16↑	[99]	miR-146↓	[101]	let-7b↑	[22]	miR-21↑	[28]	miR-21↓	[28]
miR-32↑	[46]	miR-137↓	[115]	miR-4677↑	[67]	miR-212↑	[38]	miR-124↓	[49]
let-7b↑	[22]	miR-19a↑	[116]			miR-328↑	[33]	miR-150↓	[64]
miR-6501↑	[113]	miR-183↑	[113]					let-7b↓↑	[91]
miR-144↓	[117]	miR-207↑	[118]					miR-146a↓↑	[106]
miR-618↑	[113]	miR-212↑	[38]					miR-155↑	[90]
miR-98↑	[47]	miR-326↑	[119]					miR-16↑	[99]
miR-155↑	[90]	miR-328↑	[33]					miR-107↑	[120]
miR-183↓	[113]	miR-335↑	[121]					miR-26b↑	[120]
miR-29a↑	[22]	miR-409↑	[122]					miR-29b↑	[120]
miR-1246↑	[123]	miR-434-3p↑	[124]						
miR-499↑	[28]	miR-467↑	[125]						
miR-21↑	[28]	miR-33↓	[126]						
		miR-34c↓	[127]						
		miR-129-3P↓	[128]						
		miR-133a↓	[33]						
		miR-448↓	[129]						
		miR-142-5p↓	[123,130]						
		miR-144↓	[117]						

### 3.2 MicroRNAs as a regulator of cell protein ACE2

The blockage of ACE2 can be accomplished through either the facilitation of its enzymatic function or the suppression of its transcription [37]. It is noteworthy that ACE2 is present in various organs, containing the heart, kidneys, blood vessels, central nervous system, digestive system and lungs [38]. Ahmed et al. have pointed out that ACE2 is found in cardiomyocytes and that attending heart-related ailments might result in increased ACE2 production [39]. The significance of ACE2 in SARS-CoV stems from the reality that the receptor binding domain (RBD) located on the S1 glycoprotein effects on ACE2 receptors [37][Fig. 1]. This reaction, especially within endothelium, could potentially resulted to neurovascular destruction because, in addition to endothelium, ACE2 is also produced in various parts of the brain [40]. In Singh et al.'s review, it was mentioned that specific microRNAs can regulate the expression of ACE2. For instance, miR-155 and miR-145 have been demonstrated to increase ACE2 gene expression, whereas miR-132,miR-19b, miR-181, miR-29, and miR-212 have been found to decrease it [38]. In their work, Gambardella et al. have evaluated the exosomal miR-145 and miR-885 in COVID-19 patients based on the hypothesis of their regulatory role on thromboembolic complications of this disease [41]. They observed that exosomal miR-145 and miR-885 have major correlation with D-dimer levels. Totally, their findings indicate that exosomal miR-885 and miR-145 are essential for thromboembolism in COVID-19 [41]. Nevertheless, specific microRNAs, like miR-125b and miR-18 in the kidneys, miR-146a in the heart, and miR-4262 in the lungs, are known to participate in the ACE2 regulation [38]. Prior research has explored the involvement of microRNAs in the cellular entry of SARS-CoV-2. For instance, miR-200c can decrease disease risk by preventing the ACE2 activity [42]. The study by Nersisyan et al. has previously elucidated the downregulation of ACE2 production by miR-141, miR-125a, and members of the miR-200 family [43]. Additionally, Eyileten et al. have pointed out that the most effective microRNAs regulating ACE2 and coagulation-related interaction networks include miR-155, miR-27a, miR-16, and let-7b. They found that the expression of these microRNAs in COVID-19 patients were lower than control groups [44].

### 3.3 MicroRNAs as a regulator of cell protein TMPRSS2

Previously, it has been documented that TMPRSS2 has a crucial role in the ACE2 cleavage and the S-protein, facilitating viral entrance using cell membrane. TMPRSS2 becomes active due to autocatalysis and, in this form, interaction with the ACE2 receptor leads to cleavage [45]. Genetic variations in TMPRSS2 may impact susceptibility to viruses. Researchers has been shown the elevated rs8134378 polymorphism of TMPRSS2 in males and promote membrane fusion and infection rate of viruses like H7N9 and H1N1 [40]. In the context of potential microRNA candidates, Ahmed et al. suggested that miR-98 could potentially modulate the production of TMPRSS2 in human endothelium [46]. Additionally, several other microRNAs, such as miR-21, miR-98, and miR-32, may have a role in suppressing the synthesis of TMPRSS2 [46,47].

### 3.4 MicroRNAs against NRP1

In previous studies, in addition to ACE2, NRP1 has been suggested to potentially play a role in COVID-19 infection by serving as a cellular intermediate for the virus's entry into the cells [48]. Katopodis et al. identified 69 microRNAs with high binding capability to NRP1 [48]. Also, Naidoo et al. indicated that some microRNAs may interact and reduce NRP1 production. These microRNAs include miR-214-3p, miR-320(a-d), miR-206, miR-124-3p, miR-24-3p, miR-199a-5p, miR-141-3p, and miR-130a-3p. They also noted that miR-204 was increased in COVID-19 patients [49]. NRP1 can also effects on cytokine overproduction in COVID-19 patients [50].

### 3.5 MicroRNAs, inflammation, and cell death pathways in COVID-19

Excessive inflammation is a defining feature of severe COVID-19 cases and is associated with the release of pro-inflammatory cytokines, known as a "cytokine storm." MicroRNAs have been shown to influence the expression of genes associated with both pro-inflammatory and anti-inflammatory responses. For example, miR-155 enhances pro-inflammatory cytokine expression, while miR-146a inhibits the inflammatory response [51,52]. Dysregulation of these microRNAs may contribute to the uncontrolled inflammation observed in severe cases. Cell death pathways, such as apoptosis and pyroptosis, are integral to the host's antiviral response. They help to limitation of replication and spread of viruses. *SARS-CoV-2* appears to manipulate these pathways to evade the immune system. Researchers have identified specific microRNAs, such as miR-34a and miR-223, that can modulate these pathways. MiR-34a enhances apoptosis in infected cells, limiting viral replication, while miR-223 has been associated with inhibiting pyroptosis and mitigating excessive inflammation [12,[53], [54], [55], [56]].

### 3.6 MicroRNAs and antiviral immune response in COVID-19

MicroRNAs influence various aspects of the immune response to viral infections. They can modulate the expression of genes involved in both innate and adaptive immune systems. In COVID-19, microRNAs have emerged as significant contributors to the host's defense mechanisms against SARS-CoV-2. In the early stages of COVID-19 disease, innate immunity is crucial in recognizing and responding to the virus. MicroRNAs, such as miR-146a and miR-155, play pivotal roles in regulating the expression of essential immune response genes. MiR-146a, for example, acts as a negative regulator of the inflammatory response by targeting molecules in the Toll-like receptor (TLR) and NF-κB signaling pathways [30,57,58]. Conversely, miR-155 promotes the expression of pro-inflammatory cytokines, contributing to the immune response against the virus [[59], [60], [61], [62]].

The adaptive immune response, characterized by producing specific antibodies and memory T-cells, is essential for long-term protection against SARS-CoV-2. MicroRNAs have been implicated in regulating the differentiation and function of T-cells and B-cells. For instance, miR-150 has been shown to impact T-cell development, while miR-155 influences B-cell responses [[62], [63], [64]]. In addition to microRNA effects on antiviral immune response, some microRNAs can directly act on *SARS-CoV-2* genome and neutralize it.

### 3.7 MicroRNAs role in obesity

Obesity is acknowledged as one of the most prevalent underlying health conditions linked to COVID-19, with a prevalence rate between 30 % and 60 % [65]. Despite extensive research conducted at molecular, biochemical, and clinical levels, the precise relationship between obesity and COVID-19 remains unclear [66,67]. Considering that susceptibility to infection varies depending on the viral lineage, it is noteworthy that the *SARS-CoV-2* virus can invade adipocytes and alter their metabolic processes, with a greater impact observed in visceral adipose tissue than subcutaneous tissue [68]. Adipose tissue serves not only as a reservoir for lipids in the human body but also as an effective component with roles in endocrine, paracrine, and autocrine functions. Fatness is characterized as an state of low-grade inflammation, and adipose tissue generates a variety of pro-inflammatory cytokines, include IL6. Furthermore, adipose tissue generates free vesicles released into the bloodstream, facilitating communication with other tissues [68,69]. Among these vesicles contents we can see various classes of microRNA molecules. The profile of micro RNAs in fatness has been vastly investigated even prior to the emergence of SARS-CoV-2, utilizing both human samples and animal models [70]. In the COVID-19 infection, one avenue of studies has centered on ACE2, the primary target for the entry of the *SARS-CoV-2* virus into cells, and the related modulatory microRNAs. Elemam et al. evaluated COVID-19 patients to assess the amounts of free ACE2 (sACE2) and four microRNAs (miR-3909, miR-421, miR-4677-3p, and miR-212-p), which serve as upward ACE2 regulators [67]. In this study, the participants were categorized based on their body mass index (BMI) into three groups: obese, overweight, and normal BMI. Increased amounts of soluble ACE2 (sACE2) were observed in all test groups compared to healthy controls. Furthermore, the obese group had higher sACE2 levels compared to the overweight group, which is consistent with prior discoveries unrelated to COVID-19. Among the analyzed microRNAs (miR-3909, miR-421, miR-212-p, and miR-4677-3p), three of them (miR-4677-3p, miR-3909, and miR-421) exhibited upregulation in all evaluated groups, while miR-212-p was increased solely in the normal BMI group. Additionally, the levels of miR-212-p were affected by disease severity and sex. It is important to note that this study is distinctive in its design, and the authors acknowledged certain limitations, such as the exclusion of patients with acute COVID-19 and the absence of consideration for the possible use of ACE inhibitors. Conversely, Bellae Papannarao et al. recognized that the increase of miR-200c might affect the susceptibility to COVID-19 in obese individuals [65]. They demonstrated an increase in the levels of miR-let-7b and miR-200c in individuals with obesity. Correspondingly, ACE2, a straight target of these microRNAs, exhibited remarkable downregulation. However, it is worth noting that studies have indicated that the inhibition of ACE2 itself does not lead to a reduction in the severity of COVID-19. It may even result in a following induction in angiotensin II levels, potentially worsening the disease. Since microRNAs are among the first epigenetic modulators, researchers evaluated free miR-200c as a potential diagnostic molecule for acute COVID-19. Recently, Roganovic proposed that a deficiency in miR-146a might contribute to developing acute COVID-19 in individuals with obesity, diabetes, or hypertension [71]. As part of the defensive reaction triggered by a virus, miR-146a is among the earliest microRNAs to be activated. It is well-established as a key regulator downstream in the Toll-like receptor (TLR) signaling pathway. Furthermore, some researchers have identified the *SARS-CoV-2* genome as a target for this specific microRNA. At numerous levels, the role of miR-146a is to inhibit an overly aggressive immune response. The observed decrease of miR-146a in fatness, as well as in individuals with diabetes and hypertension, may explain the link between these conditions and the manifestation of more severe forms of the disease and poorer outcomes. In her paper, Mormile analyzed acute COVID-19 cases in obese adolescent patients and emphasized the crucial effect of miR-126 downregulation [72]. It has been appointed that in the fatness, visceral lipid tissue exhibits functional abnormalities, including a reduction in the miR-126 levels in extracellular vehicles (EVs). The decrease of miR-126 level can impact *SARS-CoV-2* infection in various ways, the most significant being an increased risk of acute respiratory distress syndrome. A recent finding underscores the new system of lung alveolar recovery by delivering miR-126 via EVs and exosomes. Both adult forms of the miR-126 products, namely miR-126-5p and miR-126-3p, are effective in the renewal of alveolar epithelium, with increasing amounts of miR-126-3p correlated to age in an age-related manner. Another mechanism contributing to its downregulation is linked to miR-126's role as an inhibitor of generating various pro-inflammatory molecules, such as IL6. Consequently, the downregulation of miR-126 in *SARS-CoV-2* infection can lead to a cytokine storm. The third system operates through the penetration of miR-126 in thrombosis occurrence, where free miR-126 decreases blood clotting tendencies. This is achieved by post-transcriptionally regulating the expression of tissue factor (TF) in extracellular vesicles. As a result, the downregulation of miR-126 increases the predisposition to thrombotic events.

### 3.8 MicroRNAs role in neurological diseases

Five years have passed since the onset of the COVID-19 pandemic, and many researches have consistently shown that individuals with neurological conditions are at an increased risk of experiencing more intensive symptoms following COVID-19 disease. Patients with autoimmune diseases like multiple sclerosis (MS) and those with degenerative diseases such as Parkinson's and Alzheimer's disease have been found to face different outcomes and higher fatality rates [73]. Similar patterns have been observed, albeit with less available data, in individuals with infrequent neurodegenerative disorders like amyotrophic lateral sclerosis/frontotemporal dementia (ALS/FTD) and prion diseases [74]. Infections caused by viruses, bacteria, or other pathogens in the nervous system have demonstrated the potential to initiate or expedite neurodegeneration and neuroinflammation, leading to various clinically significant symptoms [75,76]. Many of these conditions are influenced by age, indicating that the aging brain is more susceptible to autoimmune destruction triggered by a cytokine storm [77]. Moreover, intensive cases of COVID-19 have been declared predominantly in aged individuals, who are formerly at an elevated danger of expanding neurological diseases, even without COVID-19 [78]. It is important to consider that viral infections may initiate preclinical stages of basic physiological and molecular neurodegenerative activities [79]. Another facet of the connection between COVID-19 and neurological disorders becomes evident when considering the wide array of neurological symptoms that occur within and after the critical phase of COVID-19. Given that *SARS-CoV-2* infection is recognized as a multi-organ disease, it can potentially affect various parts over an extended course, including the central nervous system [80]. The chronic, progressive, and acute effects on the CNS due to the COVID-19 disease result from the combined impact of various systems associated with the virus's life cycle. As mentioned earlier, the presence of the ACE2 receptor is notable in the CNS, exclusively in regions like the pons and medulla oblongata within the brain structures, as well as the visual areas and choroid plexus of the occipital lobe [81]. Furthermore, it is worth noting that the S1 spike protein exhibits several features reminiscent of prions, particularly the ability to promote the unusual and gradual accumulation of misfolded proteins [82]. This characteristic aligns with numerous case reports highlighting the co-occurrence of COVID-19 and prion diseases in patients [[83], [84], [85]]. Another commonly observed neurological outcome is anti-NMDA encephalitis, documented in adult and children COVID-19 patients [86]. There is also a potential overlap with prion diseases in some cases [87]. The initial clinical signs of most neurodegenerative diseases share common features, and by the time these symptoms become apparent, the underlying molecular changes are already well underway, often reaching a stage where they cannot be reversed or halted. There is a significant focus on developing effective biomarkers for COVID-19, and this intersects with the critical need for biomarkers in neurodegenerative disorders, with microRNAs emerging as promising candidates. As mentioned earlier, studies have revealed changes in the levels of certain microRNAs in the serum of COVID-19 patients, suggesting their potential utility as markers for identifying between the acute and post-acute phases of the disease [22]. It is worth noting that certain microRNAs exhibit similar changes in expression both in neurological disorders and during COVID-19, suggesting the possibility of using them as biomarkers to identify neurological complications arising from COVID-19. MiR-155 levels are elevated in COVID-19 patients and are associated with the severity of the disease [88]. Furthermore, in various neurological disorders like AD, MS, and Down's syndrome dementia, the increased expression of this pro-inflammatory microRNA contributes to heightened inflammation within the central nervous system (CNS). Domenica Zingale et al. at their review had checked the MiR-155 pro-inflammatory as an important regulatory role [89]. This occurs by targeting several anti-inflammatory regulators, leading to increased blood-brain barrier permeability, activation of T lymphocytes, and the formation of beta-amyloid plaques [89,90]. Let-7b is another microRNA that exhibits elevated expression in *SARS-CoV-2* infections and individuals with neurodegenerative diseases like PD and AD. Huang et al. have evaluated the PD mice and cell line models for the effects of let-7b-5p on cell apoptosis. They demonstrated that let-7b-5p contributes to cell apoptosis in PD by targeting HMGA2 [91]. Zhang et al. have investigated the expression of plasma let-7 family in anti-N-methyl-D-aspartate receptor (NMDAR) encephalitis. They showed down-regulation of let-7 family microRNAs in cases of anti-NMDA encephalitis and has been suggested let-7 family members as a potential marker for identifying this status [92]. Many additional microRNAs within the same cluster, including let-7d, 7a, and let-7f, have been found to exhibit notably reduced expression levels in individuals with anti-NMDA encephalitis, and many of these microRNAs have also been linked to various other neurological conditions [92]. Wang has studied more than 50 microRNAs that display differential expression in COVID-19 and comparing them to microRNAs linked to anti-NMDA encephalitis, researchers identified seven microRNAs that are shared between these two conditions. These prevalent microRNAs are miR-21, miR-107, miR-26b, miR-29b, let-7f, let-7a, and miR-155 [93]. Conversely, there is a decrease in the serum levels of several microRNAs in both COVID-19 and neurological diseases. The reduced expression of miR-21, which has an anti-inflammatory role by targeting pro-inflammatory genes, results in a pro-inflammatory outcome in *SARS-CoV-2* infections, exacerbating the disease's intensity. This downregulation also impacts neuroinflammation and presents a potential target for treatment [94]. Similarly, miR-31 targets various genes associated with AD, PD, and multiplex system atrophy, and its reduced production could contribute to neurodegeneration [95]. Conversely, studies examining the expression of miR-31 in *SARS-CoV-2* infections have yielded inconsistent outcomes. Keikha et al. have evaluated the relative expression of some microRNAs includes miR-31 in various grades of COVID-19 patients. They found a significant decrease of miR-31 expression in hospitalized patients in comparison to non-hospitalized patients [96,97]. Another microRNA associated with *SARS-CoV-2* infection, miR-16, is known for suppressing genes linked to neurodegenerative disorders. Interestingly, Gonzalo-Calvo et al. found a reverse correlation between the serum levels of miR-16 and the severity of COVID-19, suggesting its potential as a prognostic biomarker [98]. Moreover, miR-16 is considered a possible therapeutic target. It is worth noting that both miR-16 and miR-146 have been formerly implicated in the primary steps of prion infection, and they have also shown promise as biomarkers for certain kinds of pneumonia [99,100]. MiR-146 is a highly abundant microRNA found in the brain, and it tends to be increased in neurodegenerative disease models and following infections [101,102]. This microRNA is suggested to act as a neuroprotective agent due to its strong inhibitory effect on neuroinflammation. Liang et al. found that miR-146 is overexpression in mice, can help to reduce cognitive decline, limit the formation of beta-amyloid plaques, and enhance the removal of misfolded molecules [103]. Additionally, it is weighted as a potential clinical marker for neurodegenerative conditions, as decreased amounts of free miR-146 have been observed in patients with Alzheimer's disease (AD) [101]. Donyavi et al. have evaluated the expression of some microRNAs in COVID-19 patients and healthy control group. They found the various and significant expression of miR-146 in healthy control group, COVID-19 and post-acute COVID-19 patients [22]. As the focus has shifted from the acute phase of COVID-19 to its chronic outcomes, there has been a growing interest in the correlation with chronic fatigue [104]. Chronic Fatigue Syndrome (CFS) is a complex status with diverse presentations and mostly overlaps with other neurological diseases, potentially sharing equivalent underlying mechanisms [105]. Many microRNAs that show differential expression in CFS are also involved in regulating the immune response. Specifically, miR-146a, miR-558, miR-124, miR-143, and miR-150 are straight linked to genes related to inflammation [[106], [107], [108]]. The similarities between CFS and long-term COVID-19 may expedite further research in uncovering the usual pathological pathways that were previously unknown.

This review has summarized findings on the distinct roles of microRNAs in COVID-19 across various groups, including patients with obesity, and those with neurological disorders. These findings have been compiled into Table 1 for easy reference. Despite the apparent differences in obesity and neurological diseases as distinct metabolic and physiological conditions, there is a possibility that they share common molecular pathways and involve the same microRNAs. Notably, miR-146a is the only microRNA that shows altered expression across all these observed conditions. These microRNAs can regulate various biological processes, such as immune responses and inflammation. As a result, they could serve as informative biomarkers and potential therapeutic targets for multiple medical conditions.

### 3.9 MicroRNAs role in diabetes and heart disease

Using microRNA in individuals with COVID-19 and diabetes may improve immune reactions within the diabetic heart, potentially alleviating cardiomyopathy and reducing the risk of heart failure [109]. Another promising approach in microRNA therapeutics involves targeting the formation of inflammasomes and cell death processes, which could benefit patients dealing with diabetes and COVID-19 [110]. In this way, microRNA can play a multifaceted role in inhibiting viral infection and lessening its adverse structural changes. Moreover, it can potentially serve as a valuable marker for diabetic individuals who have contracted COVID-19. MicroRNA holds promise as both a biomarker and a therapeutic option for individuals dealing with diabetes and COVID-19. In diabetes, variations in the production of free miroRNAs may serve as valuable biomarkers to assess the intensity of COVID-19, both in cases with and without cardiac malfunction. Diabetes also elevates the risk of thrombosis while decreasing the levels of cardioprotective microRNAs like miR-133a in the heart [111]. Utilizing microRNA mimics to increase cardioprotective microRNA levels has shown potential in mitigating heart failure in diabetic patients.

In the context of *SARS-CoV-2* infection, the virus enters host cells via the angiotensin-converting enzyme 2 (ACE2). Once inside the lung cell, the virus replicates to produce more viral particles. MicroRNA can be crucial in constraining viral replication, enhancing the immune system's response, and preventing lung deterioration. This, in turn, can potentially improve cardiovascular outcomes [112]. Consequently, microRNA emerges as a promising candidate for therapeutic intervention and biomarker assessment in individuals with diabetes and COVID-19. In the context of COVID-19 and its effect on the heart, particular attention is given to specific microRNAs because of their potential influence on *SARS-CoV-2* protein production and heart failure. Notably, miR-15b-5p exhibits reduced levels with age in individuals with coronary artery disease, and miR-30e-3p shows a similar age-related decrease in cases of myocardial hurt. Both of these microRNAs can target the *SARS-CoV-2* genome. Lipotoxicity and metabolic change are substantial factors in diabetic heart failure, and microRNAs involved in these routes might play crucial roles in patients with COVID-19 and diabetes. Mishra et al., indicates that elevating cardiac miR-133a levels in diabetic hearts can reduce cardiac lipid accumulation, suggesting a potential role for miR-133a in regulating lipotoxicity and metabolic remodeling in diabetic hearts [33]. Moreover, it targets angiotensinogen, potentially influencing ACE2 receptor function in congestive heart failure. Additionally, miR-133a has implications for controlling arrhythmia, as it modulates electrical repolarization resulting from pressure overload in the heart [111]. MiR-133a is abundant in the human heart and is downregulated in diabetic and non-diabetic heart failure. It is essential for adult heart function, and its loss can lead to cardiac hypertrophy and dysfunction. Importantly, overexpressing miR-133a supports the heart against cardiac fibrosis due to pressure extra load and spoiled contractility resulting from diabetes. Therefore, miR-133a is a promising candidate for evaluating its role in heart failure among patients with COVID-19 and diabetes. Additionally, several other microRNAs, including miR-21, miR-1, miR-328, miR-208, miR-590, and miR-212, are implicated in arrhythmia, and their specific targets and function in arrhythmia and cardiac conductance have been newly elucidated [33].

## 4 Conclusions

This review has highlighted the functional connection between microRNA regulation in *SARS-CoV-2* infection and the conditions of obesity and neurological disorders. Therefore, studying the circulating microRNA profile at various stages of COVID-19 and categorizing patients based on their microRNA status could offer valuable clinical insights, guiding potential interventions in the future. Emphasizing the significance of assessing molecular alterations, not just during the initial disease phase but also post-contagion, recent research is concentrated on evaluating the heightened risk of enduring health issues following the acute phase of the disease. The goal is to find effective biomarkers for tailored prevention strategies. We have compiled research highlighting microRNAs as suitable biomarkers for enabling personalized monitoring of disease progression. We conclude that early identification of individuals with a heightened risk of COVID-19 complications, as well as the development of chronic and advancing conditions, offers the prospect of more effective treatments during potentially reversible stages of the disease. Furthermore, the review assesses the advancements achieved so far and the challenges that need to be overcome using microRNA biomarkers, especially when combined with other metabolic indicators. The future trajectory of microRNA research should emphasize the role of microRNAs within a profile of various biomarkers rather than seeking a singular "perfect" biomarker. Additionally, developing technologies that offer standardized and straightforward laboratory methods will be crucial for their clinical implementation.

## Funding

This study did not receive dedicated funding from public, commercial, or nonprofit organizations.

## 5 Informed consent statement

Not applicable.

## CRediT authorship contribution statement

**Abdollah Kebriaei:** Writing – review & editing, Data curation, Conceptualization. **Reza Besharati:** Resources, Methodology. **Hasan Namdar Ahmad Abad:** Methodology, Investigation, Data curation. **Shahrzad Havakhah:** Resources, Methodology, Formal analysis. **Mahsa Khosrojerdi:** Writing – original draft, Visualization, Resources. **Amir Azimian:** Writing – review & editing, Writing – original draft, Validation, Supervision, Methodology, Data curation, Conceptualization.

## Declaration of competing interest

The authors declare that they have no known competing financial interests or personal relationships that could have appeared to influence the work reported in this paper.
